# Feasibility study of docetaxel plus bevacizumab as first line therapy for elderly patients with advanced non-small-cell lung cancer: Thoracic Oncology Research Group (TORG) 1014

**DOI:** 10.1186/s12885-015-1756-1

**Published:** 2015-10-19

**Authors:** Yusuke Takagi, Yukio Hosomi, Fumihiro Oshita, Hiroaki Okamoto, Nobuhiko Seki, Koichi Minato, Hiromi Aono, Kouzo Yamada, Yusuke Okuma, Naoya Hida, Takahiko Sakamoto, Yosuke Miura, Makiko Yomota, Akira Satoh, Hideo Kunitoh, Kentaro Sakamaki, Masahiko Shibuya, Koshiro Watanabe

**Affiliations:** 1 Department of Thoracic Oncology and Respiratory Medicine, Tokyo Metropolitan Cancer and Infectious Diseases Center, Komagome Hospital, 3-18-22, Honkomagome, Bunkyo-ku, Tokyo 113-8677 Japan; 2 Kanagawa Cancer Center, 2-3-2 Nakao, Asahi-ku, Yokohama 241-8515 Japan; 3 Yokohama Municipal Citizen’s Hospital, 56 Kazawa-cho, Hodogaya-ku, Yokohama 240-8555 Japan; 4 Teikyo University School of Medicine, 2-11-1 Kaga, Itabashi-ku, Tokyo 173-8606 Japan; 5 Gunma Prefectural Cancer Center, 617-1 Takahayashi-Nishicho, Ota City, Gunma 373-8550 Japan; 6 Mitsui Memorial Hospital, 1 Kanda-Izumicho, Chiyoda-ku, Tokyo 101-8643 Japan; 7 Japanese Red Cross Medical Center, 4-1-22 Hiroo, Shibuya-ku, Tokyo 150-8935 Japan; 8 Kyoto University Graduate School of Medicine, Yoshida-Konoecho, Sakyo-ku, Kyoto 606-8501 Japan

**Keywords:** Bevacizumab, Docetaxel, Elderly patients, Feasibility study, Non-small-cell lung cancer

## Abstract

**Background:**

Docetaxel monotherapy is one of the standard treatments for non-small-cell lung cancer in elderly patients. The addition of bevacizumab to docetaxel seems promising; however, the feasibility of this combination has not been investigated in such patients.

**Methods:**

Patients with advanced non-squamous non-small-cell lung cancer aged 70 years or older who had not previously received cytotoxic chemotherapy were enrolled. Patients in the Level 0 cohort received docetaxel 60 mg/m^2^ and bevacizumab 15 mg/kg, whereas those in the Level-1 cohort received docetaxel 50 mg/m^2^ and bevacizumab 15 mg/kg. Chemotherapy was repeated 3 weekly for six cycles. The primary endpoint was toxicity and the secondary endpoints were response rate, progression-free survival, overall survival, and proportion of patients who underwent three or more cycles of chemotherapy.

**Results:**

Twenty-one patients were enrolled from December 2010 to September 2012 at six institutes. Of the nine patients enrolled in Level 0, two experienced dose-limiting toxicity (febrile neutropenia and prolonged Grade 4 neutropenia in one patient, and Grade 3 infection in another patient) during the first cycle. Enrollment to the Level 0 cohort was terminated because two patients developed Grade 4 sepsis during later cycles. The remaining 12 patients were enrolled in the Level-1 cohort, in which two dose-limiting toxicities (prolonged Grade 4 neutropenia and Grade 3 increased aminotransferase level) were observed. No patient in the Level-1 cohort experienced Grade 4 nonhematologic toxicity. Grade 4 neutropenia occurred in 89 % of Level 0 patients and 50 % of Level-1 patients. The proportion of patients who experienced Grade 3/4 infection, febrile neutropenia or sepsis was 44 % in the Level 0 cohort, and 8 % in the Level-1 cohort. The overall response rate to chemotherapy and progression-free survival were 29 % (95 % CI, 11–52 %) and 5.9 months (95 % CI, 3.6–9.1 months), respectively. Efficacy outcomes did not differ significantly between the cohorts.

**Conclusions:**

Toxicities were tolerable in level-1 cohort. The recommended dose of combination chemotherapy with docetaxel and bevacizumab for elderly patients was determined as 50 mg/m^2^ of docetaxel and 15 mg/kg of bevacizumab and toxicities were tolerable. Further studies are warranted.

**Trial registration:**

UMIN Clinical Trial Registry; UMIN000004240.

## Background

Lung cancer is the leading cause of cancer-related mortality, with over 1.3 million people dying annually from lung cancer worldwide [1]. In Japan, approximately two thirds of cases of newly diagnosed lung cancer occur in patients aged greater than 70 years [2], and the proportion of older patients with lung cancer continues to increase [3]. Older patients tend to have reduced bone marrow and hepatic and renal function compared to younger patients, which is likely a key determinant in the potential for increased adverse effects in response to anticancer chemotherapies [4]. Since studies have shown that older patients with NSCLC have higher rates of toxicities and complications related to treatment with platinum drugs [5, 6], the standard treatment approach for older patients may be different than that for younger patients.

A randomized trial comparing vinorelbine with best supportive care (the Elderly Lung Cancer Vinorelbine Italian [ELVIS] study) [7] and another three-arm trial evaluating vinorelbine, gemcitabine, and a combination of those two drugs (the Multicenter Italian Lung Cancer in the Elderly [MILES] study) [8] have demonstrated that monotherapy is effective in elderly patients with NSCLC. The West Japan Thoracic Oncology Group (WJTOG) 9904 study found that docetaxel monotherapy results in a trend toward longer survival compared with vinorelbine monotherapy in patients aged 70 years or older with advanced NSCLC [9]; this finding has been supported by a similar study conducted by the Hellenic Oncology Research Group [10]. In elderly patients, carboplatin-paclitaxel resulted in significantly longer overall survival (OS) than vinorelbine or gemcitabine in the recent Intergroupe Francophone de Cancérologie Thoracique (IFCT) 0501 trial [11]. The current standard therapy for elderly patients with NSCLC is platinum doublet. Since a direct comparison between docetaxel and carboplatin-paclitaxel has not been conducted in the setting of a large randomized trial and a Japanese randomized phase 2 trial did not show a significant difference in OS [12], docetaxel monotherapy remains an optional treatments for NSCLC in elderly patients.

Bevacizumab, an anti-vascular endothelial growth factor antibody, exerts antitumor activity by inhibiting angiogenesis [13] and by improving drug delivery by lowering the interstitial pressure within the tumor [14]. The Eastern Cooperative Oncology Group (ECOG) demonstrated a significant survival advantage with the addition of bevacizumab to carboplatin-paclitaxel in a randomized phase III trial [15]. However, a large retrospective study [16] and a subset analysis of the ECOG 4599 study [17] have shown no significant benefit in adding bevacizumab to carboplatin-paclitaxel in patients aged ≥65 years. Despite these findings, the value of incorporating bevacizumab into chemotherapy for older patients remains unclear due to the lack of prospective randomized trials with sufficient statistical power. In our study, we seek to further investigate the combination of docetaxel and bevacizumab in an elderly patient population with NSCLC. The goal of this multi-center study is to evaluate the tolerability of the combination and to establish the appropriate dosage in elderly patients with NSCLC.

Although the toxicity profile of bevacizumab differs between older and younger patients [16], no study has evaluated the tolerability of docetaxel-bevacizumab in older patients. Therefore, the goal of this multi-center study was to evaluate this combination’s tolerability and to determine the appropriate dosage in older patients with NSCLC.

## Methods

This multi-center, open-label, dose-finding study was conducted in accordance with the Declaration of Helsinki and the Ethical Guidelines for Clinical Research issued by the Japanese Ministry of Health, Labour and Welfare. The protocol was approved by the Clinical Trial Review Committee of the Thoracic Oncology Research Group and the Institutional Review Board or Ethics Committee of each participating center. All patients provided written informed consent. The clinical trial registry number was UMIN000004240.

### Study participants

Eligible patients were aged ≥70 years and had stage IIIB/IV or recurrent non-squamous NSCLC. No patient had received prior cytotoxic chemotherapy; however, prior treatment with an epidermal growth factor receptor tyrosine kinase inhibitor (EGFR-TKI) was allowed. Uracil-tegafur therapy as adjuvant chemotherapy for Stage I NSCLC was also allowed. Additional inclusion criteria were ECOG performance status (PS) of 0 or 1, at least one measurable focus of disease, neutrophil count ≥2,000 cells/μL, hemoglobin ≥9.5 g/dL, platelet count ≥100,000 cells/μL, aspartate aminotransferase and alanine aminotransferase (ALT) levels ≤2.5× the upper limit of normal, total bilirubin level ≤1.5× the upper limit of normal, serum creatinine level ≤1.2 mg/dL, oxygen saturation by pulse oximetry ≥93 %, proteinuria ≤1+, and life expectancy longer than 12 weeks.

Patients were excluded if they had undergone any surgery within the past 8 weeks, exploratory thoracotomy within the last 4 weeks, or radiotherapy or minor procedures (for example, fluid drainage with a chest tube) within the last 2 weeks before enrollment. Other major exclusion criteria were a history of hemoptysis, a history of peptic ulcer within the past year, severe or uncontrollable comorbidities, brain metastases, massive pleural/pericardial effusion or ascites, regular use of anticoagulants (≤325 mg per day of aspirin was permitted), or concomitant malignancy.

### Procedures

Eligible patients were given docetaxel and bevacizumab intravenously and the treatment was repeated every 3 weeks. Patients in the Level 0 cohort received docetaxel 60 mg/m^2^ (the recommended dose for monotherapy in Japan) and bevacizumab 15 mg/kg, and those in the Level -1 cohort received docetaxel 50 mg/m^2^ and bevacizumab 15 mg/kg. In each cohort, if less than four of the first six patients had experienced dose-limiting toxicity (DLT), an additional six patients were to be enrolled in the same cohort. If four or more of the first six patients in the Level 0 cohort had experienced DLT, the study was to proceed to Level -1. If four or more of the first six patients in the Level-1 cohort had experienced DLT, the study was to be terminated. Overall toxicity was evaluated after the treatment of 12 patients in a given cohort. If six or more of the 12 patients in the Level 0 cohort had experienced DLT, the study was to proceed to Level-1.

DLT was defined as the following toxicities occurring in the first cycle: Grade 4 neutropenia lasting 4 days or more, febrile neutropenia, Grade 4 thrombocytopenia, non-hematological toxicities ≥ Grade 3 (except for nausea, hyponatremia, weight loss, anorexia, infusion reaction, and hypertension), or Grade 4 hypertension. The recommended dose for future study was determined by the overall toxicities, including all of the adverse events occurring during treatment. Primary prophylactic use of granulocyte colony-stimulating factor (G-CSF) was not allowed. G-CSF administration was recommended for patients with Grade 4 neutropenia or Grade 4 leukopenia, this being determined by the availability of coverage by Japanese public health insurance. Although this trial defined study treatment as six cycles of docetaxel plus bevacizumab, any subsequent therapies, including continuation of docetaxel and/or bevacizumab, were allowed.

### Assessments

Toxicities were evaluated according to the National Cancer Institute’s Common Toxicity Criteria for Adverse Events, version 4.0. Complete blood counts and serum chemistries were obtained at the beginning of each cycle, and weekly during the first cycle.

Tumor response to the chemotherapy was assessed using the Response Evaluation Criteria in Solid Tumors, version 1.1. After baseline evaluation, tumor status was assessed every 6 weeks (two cycles). Two consecutive evaluations were required to confirm complete response or partial response to the chemotherapy.

### Statistical methods

The primary objectives were to evaluate the toxicity profile and to determine the recommended dose of docetaxel-bevacizumab therapy in patients aged 70 years or older with non-squamous NSCLC. The secondary endpoints were the response rate to chemotherapy, progression-free survival (PFS), OS, and the proportion of patients who underwent three or more cycles of chemotherapy. PFS was defined as the number of months between enrollment and date of disease progression or death. Patients who remained alive without disease progression at the end of follow-up, and patients who had started subsequent chemotherapy without disease progression, were censored. OS was defined as the number of months between study enrollment and date of death. Patients alive at the end of follow-up were censored. Differences in proportions between the cohorts were evaluated using the χ^2^ test. Differences in age were compared using the Student’s *t*-test. PFS and OS were estimated using the Kaplan–Meier method, and the log-rank test was used for inter-group comparisons. All tests were two-sided with a significance level of 0.05.

## Results

### Patient characteristics

From December 2010 to September 2012, 21 patients with stage IIIB/IV or recurrent non-squamous NSCLC were enrolled at six institutes. Nine patients were enrolled in the Level 0 cohort (docetaxel 60 mg/m^2^, bevacizumab 15 mg/kg) and subsequently 12 were enrolled in the Level-1 cohort (docetaxel 50 mg/m^2^, bevacizumab 15 mg/kg). The median age was 75 years (range, 70–84 years), and 12 patients (57 %) were female. Most patients had adenocarcinoma (90 %) and stage IV disease (86 %). EGFR mutations were detected in four patients (19 %). ECOG-PS was zero in eight patients (38 %) and one in 13 patients (62 %). Three patients (14 %) had received prior EGFR-TKI therapy; all three had an EGFR-activating mutation. One patient (5 %) had undergone adjuvant chemotherapy with uracil-tegafur after complete resection of Stage I NSCLC. Baseline characteristics did not differ significantly between the two cohorts (Table 1).

**Table 1:** Patient characteristics

Category	Subcategory	Level 0, *N*	Level -1, *N*
Total		9	12
Median age (range), yr		78 (74–79)	75 (70–84)
Sex	Female	5	7
	Male	4	5
Histological type	Adenocarcinoma	8	11
	NSCLC, NOS	1	1
Disease stage	IIIB	1	1
	IV	8	10
	Recurrent	0	1
Performance status	0	3	5
	1	6	7
EGFR mutation	Ex19 del	0	2
	L858R	2	0
	Wild type	5	7
	Unknown	2	3

*EGFR*: Epidermal growth factor receptor, *Ex*19*del*: Exon 19 deletions, *NOS*: Not otherwise specified, *NSCLC*: Non-small-cell lung cancer

### Treatment and safety

Nine patients received cycles of 60 mg/m^2^ of docetaxel and 15 mg/kg of bevacizumab (Level 0). The median number of cycles delivered was four, 78 % of patients underwent three or more cycles. Two patients (22 %) completed six cycles of chemotherapy. The reasons for terminating chemotherapy were adverse events in five patients (56 %) and disease progression in two (22 %). One patient required dose reduction of docetaxel due to neutropenia, and five patients experienced delays in treatment. The reasons for treatment delay were administrative (e.g., public holidays) or to suit patients’ schedules, except in the case of one patient with prolonged neutropenia.

DLT, which was evaluated in the first cycle, occurred in two patients in the Level 0 cohort: one patient developed febrile neutropenia and Grade 4 neutropenia lasting for more than 4 days and the other patient had Grade 3 infection. In addition, two other patients developed Grade 4 sepsis during later cycles. The first patient with severe sepsis was a 75-year-old man who developed Grade 4 neutropenia (280 cells/μL) on day 11 of the fourth cycle and subsequently fever (39.3 °C) and septic shock on day 12. He recovered after treatment with intravenous cefepime, G-CSF, and dopamine. The second patient was a 74-year-old woman who developed Grade 4 neutropenia (100 cells/μL), fever of 40.0 °C, hypoxemia, and septic shock: blood cultures revealed *Escherichia coli* and *Klebsiella pneumoniae*. She recovered after treatment with intravenous cefepime, G-CSF, and dopamine. Due to the life-threatening events in these two cases, enrollment was stopped by the Data and Safety Monitoring Board (DSMB). After detailed examination of the treatment course of these two patients and other identified toxicities, the DSMB recommended terminating enrollment in the Level 0 cohort and continuing the trial with the Level-1 cohort.

Twelve patients were given 50 mg/m^2^ of docetaxel and 15 mg/kg of bevacizumab (Level-1). The median number of cycles delivered was four; 75 % of patients underwent three or more cycles. Three patients (25 %) completed six cycles of chemotherapy. The reasons for terminating chemotherapy were patients’ decision in four patients, adverse events in three, and disease progression in two. DLT occurred in two patients in the Level-1 cohort; one patient developed Grade 4 neutropenia lasting for more than 4 days and the other patient experienced a Grade 3 increase in the ALT level. No Grade 4 non-hematologic toxicity was observed in this cohort and thus the recommended dose for a future trial was determined as 50 mg/m^2^ of docetaxel and 15 mg/kg of bevacizumab. Four patients required delays in treatment due to prolonged neutropenia, and six patients experienced administrative delays. No treatment-related deaths occurred throughout the study.

Toxicity profiles of each cohort are shown in Table 2 (Level 0) and Table 3 (Level-1), respectively. Neutropenia and leukocytopenia were frequently observed, more patients tending to develop Grade 4 neutropenia in the Level 0 (89 %) than in the Level-1 cohort (50 %, *p* = 0.051). All patients (100 %) in Level 0 and 83 % in Level-1 developed hematological toxicities ≥ Grade 3. Grade 3/4 infection, febrile neutropenia, and sepsis were also marginally more frequent in the Level 0 cohort (44 %) compared to the Level-1 cohort (8 %, *p* = 0.051). In the Level 0 cohort, 56 % of patient developed Grade 3 nonhematological toxicities, and the proportion in the Level -1 cohort was 58 %. G-CSF was administered to 89 % of patients in the Level 0 cohort and 58 % of patients in the Level-1 cohort.

**Table 2:** Toxicities in Level 0 cohort

Toxicity	G1, %	G2, %	G3, %	G4, %
Hematologic				
Neutropenia	0	0	11	89
Leukopenia	0	11	56	33
Anemia	56	22	0	0
Thrombocytopenia	56	11	0	0
Nonhematologic				
AST increased	33	0	11	0
ALT increased	33	0	11	0
Anorexia	11	33	22	0
Stomatitis	11	22	11	0
Nausea	11	33	0	0
Diarrhea	22	22	0	0
Vomiting	22	11	0	0
Allergic reaction	22	11	0	0
Hypertension	0	11	0	0
Dyspnea	0	11	0	0
Peripheral neuropathy	44	0	0	0
Constipation	33	0	0	0
Edema limbs	22	0	0	0
Weight loss	11	0	0	0
Febrile neutropenia	-	-	11	0
Infection	0	0	11	0
Sepsis	-	-	-	22
Hypoxia	-	0	11	0

*ALT*: Alanine aminotransferase, *AST*: aspartate aminotransferase, *G*: Grade (according to the National Cancer Institute’s Common Toxicity Criteria for Adverse Events, version 4.0)

**Table 3:** Toxicities in Level-1 cohort

Toxicity	G1, %	G2, %	G3, %	G4, %
Hematologic				
Neutropenia	8	8	25	50
Leukopenia	17	25	50	8
Anemia	58	8	8	0
Thrombocytopenia	25	8	0	0
Nonhematologic				
AST increased	17	0	0	0
ALT increased	17	0	8	0
Anorexia	25	42	8	0
Nausea	42	8	8	0
Diarrhea	17	17	8	0
Vomiting	8	0	8	0
Stomatitis	25	17	0	0
Constipation	58	8	0	0
Fatigue	42	8	0	0
Hypertension	8	8	0	0
Peripheral neuropathy	8	8	0	0
Weight loss	17	0	0	0
Arthralgia	8	0	0	0
Febrile neutropenia	-	-	8	0
Intracranial hemorrhage	0	0	8	0

*ALT*: alanine aminotransferase, *AST*: aspartate aminotransferase, *G*: Grade (according to the National Cancer Institute’s Common Toxicity Criteria for Adverse Events, version 4.0)

### Efficacy

**Fig. 1:**
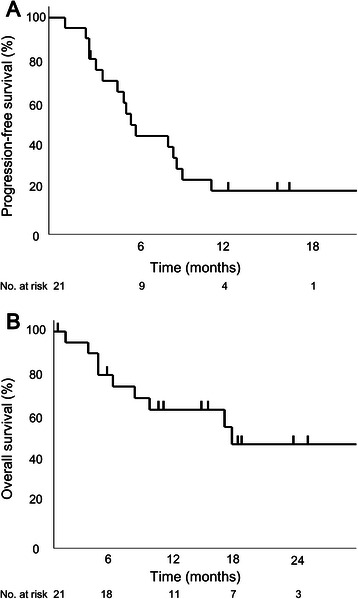
Kaplan-Meier curves for progression-free survival **a** and overall survival **b**

The overall response rate to chemotherapy was 29 % (95 % confidence interval [CI], 11–52), and the disease control rate was 71 % (95 % CI, 48–89). At a median follow-up time of 12.2 months, the median PFS and OS were 5.9 months (95 % CI, 3.6–9.1) and 18.3 months (95 % CI, 7.7 months to not reached), respectively (Fig. 1). OS was not mature: only 43 % of events had occurred at the time of analysis.

**Fig. 2:**
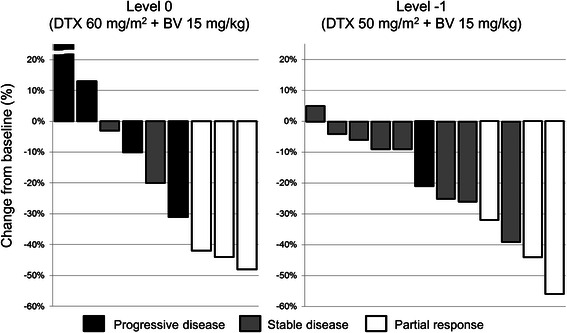
Waterfall plot of best percent change in target lesions in each cohort. BV, bevacizumab; DTX, docetaxel

For the Level 0 cohort, response rate, median PFS, and median OS were 33 %, 4.7 months, and 11.0 months, respectively, and for the Level-1 cohort, 25 %, 8.7 months, and 18.3 months, respectively. Response rates did not differ significantly between the two cohorts (Fig. 2). Although PFS and OS appeared longer in the Level-1 cohort, the differences were not statistically significant (*p* = 0.11 and *p* = 0.44, respectively).

Disease progression was observed in 17 patients. Of these, nine (53 %) underwent subsequent chemotherapy, including pemetrexed in five patients and gefitinib in two.

## Discussion

This is the first dose-finding study evaluating the combination of docetaxel/bevacizumab therapy in older patients with non-squamous NSCLC. The addition of bevacizumab to 60 mg/m^2^ of docetaxel was not tolerated from the toxicity standpoint. The older patients in our study experienced a significantly higher incidence of toxicities with the addition of bevacizumab compared to what has been noted in younger patients, which is likely a reflection of decreased organ function and increased vulnerability to infection in older patients.

Of the nine patients who received 60 mg/m^2^ of docetaxel and 15 mg/kg of bevacizumab, eight (89 %) developed Grade 4 neutropenia, four (44 %) developed severe infection or febrile neutropenia, and two (22 %) developed life-threatening sepsis. Primary prophylactic G-CSF was not allowed in this trial because only 12.5 % of patients had developed febrile neutropenia in a previous Japanese phase III trial of docetaxel therapy in older patients [9]. Even taking the absence of primary prophylactic G-CSF into consideration, the toxicities observed in the Level 0 cohort were not acceptable in older patients.

A previous Japanese phase III trial in which patients aged 70 years or older with advanced NSCLC were treated with 60 mg/m^2^ of docetaxel reported Grade 4 neutropenia in 56.8 % of patients and febrile neutropenia in 12.5 % [9]. Pre-clinical studies have shown that bevacizumab enhances the efficacy of docetaxel [16, 18]. A subset analysis of elderly participants enrolled in the ECOG 4599 study [17] showed that when bevacizumab was added, more patients tended to have severe toxicities including Grade 4 neutropenia. A meta-analysis also showed that addition of bevacizumab to cytotoxic chemotherapy was associated with a higher incidence of neutropenia ≥ Grade 3 and febrile neutropenia [16]. These findings help explain the relatively higher incidence of neutropenia and infection observed in the Level 0 cohort in this study.

By contrast, treatment with 50 mg/m^2^ of docetaxel and 15 mg/kg of bevacizumab was much better tolerated than 60 mg/m^2^ of docetaxel alone. Neutropenia and infection in the Level-1 cohort were relatively less frequent than those in historical cohort with docetaxel monotherapy [9], and no Grade 4 non-hematological toxicity occurred in Level-1 cohort. In previous IFCT-0501 study [11], the hematological toxicity of carboplatin and weekly paclitaxel regimen was similar in the present Level-1 cohort, with neutropenia (≥Grade3) and febrile neutropenia occurring in 48.4 and 9.4 % of the patients, respectively. On the other hand, they observed treatment-related deaths in 4.4 % of the patients, whereas no treatment-related deaths occurred in the present study. Although the sample size was limited because of the nature of this being a feasibility study, data from the Level-1 cohort in this trial support the feasibility of treatment with 50 mg/m^2^ of docetaxel and 15 mg/kg of bevacizumab for older patients with advanced non-small cell lung cancer.

Despite that the usual dose of docetaxel in NSCLC is 75 mg/m2 in most countries, a Japanese phase I study has determined a recommended dose of docetaxel monotherapy for NSCLC of 60 mg/m^2^, the DLT having been neutropenia [19]. The adequacy of this lower dose for Asian patients has been validated by a randomized trial in China comparing two doses [20]. Our study noted a significantly increased incidence of neutropenia with the addition of bevacizumab, which supports the notion that the addition of bevacizumab results in a lower tolerable dose of docetaxel. [16]. Further, elderly patients have poorer bone marrow function compared to younger patients and also seem to be more vulnerable to infectious diseases.

The key limitation of this study was that the recommended dose was not determined on the basis of DLTs observed in the first cycle but was instead determined by toxicities observed in later cycles. The goal of this study was to determine the appropriate dose of combination therapy with docetaxel and bevacizumab for use in future studies. Another justification for this dose is that this regimen has the potential to be adapted to daily practice. One more limitation of this study was that two populations are mixed in this trial, EGFR wild-type and EGFR-mutants. This might be a bias of the study.

The antitumor efficacy did not differ significantly between the two doses of combination docetaxel and bevacizumab chemotherapy, and equivalent to the previous studies [20]; however, the sample size was too small to compare efficacy outcomes. Additionally, because eligibility for bevacizumab is in itself a strong predictor of better prognosis [21], we are not able to draw any definite conclusions about the efficacy of these regimens in elderly patients.

## Conclusions

The recommended dose of docetaxel in combination with 15 mg/kg of bevacizumab for elderly patients was determined to be 50 mg/m^2^. Toxicities related to chemotherapy in elderly patients are often different from those seen in younger patients and warrant vigilant attention. A randomized trial comparing docetaxel plus bevacizumab with pemetrexed plus bevacizumab in elderly patients with NSCLC is ongoing.

## Abbreviations

NSCLC: Non-small cell lung cancer
OS: Overall survival
EGFR: Epidermal growth factor receptor
EGFR-TKI: Epidermal growth factor receptor tyrosine kinase inhibitor
PS: Performance status
DLT: Dose-limiting toxicity
G-CSF: Granulocyte colony-stimulating factor
PFS: Progression-free survival
DSMB: Data and safety monitoring board
BV: Bevacizumab
DTX: Docetaxel
Ex19del: Exon 19 deletions
NOS: Not otherwise specified
ALT: Alanine aminotransferase
AST: Aspartate aminotransferase
G: Grade

## References

[1] Jemal A, Bray F, Center MM, Ferlay J, Ward E, Forman D (2011). Global cancer statistics. CA Cancer J Clin.

[2] The Foundation for Promotion of Cancer Research. Cancer Statistics in Japan 2012 [Center for Cancer Control and Information Services Web site]. Jan 15, 2013. Available at: http://ganjoho.jp/en/professional/statistics/table_download.html. Accessed Dec 21, 2013.

[3] Kaneko S, Ishikawa KB, Yoshimi I, Marugame T, Hamashima C, Kamo K (2003). Projection of lung cancer mortality in Japan. Cancer Sci.

[4] Lichtman SM, Villani G (2000). Chemotherapy in the elderly: pharmacologic considerations. Cancer Control.

[5] Oshita F, Kurata T, Kasai T, Fakuda M, Yamamoto N, Ohe Y (1995). Prospective evaluation of the feasibility of cisplatin-based chemotherapy for elderly lung cancer patients with normal organ functions. Jpn J Cancer Res.

[6] Tamiya A, Tamiya M, Shiroyama T, Kanazu M, Hirooka A, Tsuji T (2013). Dose escalation study of carboplatin-pemetrexed followed by maintenance pemetrexed for elderly patients with advanced nonsquamous nonsmall-cell lung cancer. Ann Oncol.

[7] The Elderly Lung Cancer Vinorelbine Italian Study Group (1999). Effects of vinorelbine on quality of life and survival of elderly patients with advanced non-small-cell lung cancer. J Natl Cancer Inst.

[8] Gridelli C, Perrone F, Gallo C, Cigolari S, Rossi A, Piantedosi F (2003). Chemotherapy for elderly patients with advanced non-small-cell lung cancer: the Multicenter Italian Lung Cancer in the Elderly Study (MILES) phase III randomized trial. J Natl Cancer Inst.

[9] Kudoh S, Takeda K, Nakagawa K, Takada M, Katakami N, Matsui K (2006). Phase III study of docetaxel compared with vinorelbine in elderly patients with advanced non-small-cell lung cancer: results of the West Japan Thoracic Oncology Group Trial (WJTOG 9904). J Clin Oncol.

[10] Karampeazis A, Vamvakas L, Agelidou A, Kentepozidis N, Chainis K, Chandrinos V (2011). Docetaxel *vs*. vinorelbine in elderly patients with advanced non--small-cell lung cancer: a hellenic oncology research group randomized phase III study. Clin Lung Cancer.

[11] Quoix E, Zalcman G, Oster JP, Westeel V, Pichon E, Lavolé A (2011). Carboplatin and weekly paclitaxel doublet chemotherapy compared with monotherapy in elderly patients with advanced non-small-cell lung cancer: IFCT-0501 randomised, phase 3 trial. Lancet.

[12] Maemondo M, Inoue A, Sugawara S, Harada T, Minegishi Y, Usui K (2014). Randomized phase II trial comparing carboplatin plus weekly paclitaxel and docetaxel alone in elderly patients with advanced non-small cell lung cancer: north japan lung cancer group trial 0801. Oncologist.

[13] Willett CG, Boucher Y, Di Tomaso E, Duda DG, Munn LL, Tong RT (2004). Direct evidence that the VEGF-specific antibody bevacizumab has antivascular effects in human rectal cancer. Nat Med.

[14] Jain RK (2005). Normalization of tumor vasculature: an emerging concept in antiangiogenic therapy. Science.

[15] Sandler A, Gray R, Perry MC, Brahmer J, Schiller JH, Dowlati A (2006). Paclitaxel-carboplatin alone or with bevacizumab for non-small-cell lung cancer. N Engl J Med.

[16] Zhu J, Sharma DB, Gray SW, Chen AB, Weeks JC, Schrag D (2012). Carboplatin and paclitaxel with vs without bevacizumab in older patients with advanced non-small cell lung cancer. JAMA.

[17] Ramalingam SS, Dahlberg SE, Langer CJ, Gray R, Belani CP, Brahmer JR (2008). Outcomes for elderly, advanced-stage non small-cell lung cancer patients treated with bevacizumab in combination with carboplatin and paclitaxel: analysis of Eastern Cooperative Oncology Group Trial 4599. J Clin Oncol.

[18] Gerber HP, Ferrara N. Pharmacology and pharmacodynamics of bevacizumab as monotherapy or in combination with cytotoxic therapy in preclinical studies. Cancer Res. 2005;65:671–80. 15705858

[19] Yokoyama A, Kurita Y, Watanabe K, Negoro S, Ogura T, Nakano M, et al. Early phase II clinical study of RP56976 (docetaxel) in patients with primary pulmonary cancer. Docetaxel Cooperative Study Group for Lung Cancer. Gan To Kagaku Ryoho. 1994;21:2609–16. 7979421

[20] Zhang L, Lu S, Cheng Y, Hu Z, Chen Z, Chen G, et al. Different-dose docetaxel plus cisplatin as first-line chemotherapy and then maintenance therapy with single-agent docetaxel for advanced non-small cell lung cancer (TFINE study, C-TONG 0904). J Clin Oncol. 2013;31(suppl):8015.

[21] Takagi Y, Toriihara A, Nakahara Y, Yomota M, Okuma Y, Hosomi Y, et al. Eligibility for bevacizumab as an independent prognostic factor for patients with advanced non-squamous non-small cell lung cancer: a retrospective cohort study. PLoS One. 2013;8:e59700. 10.1371/journal.pone.0059700 PMC3608561 23555751

